# *Melanophilin* regulates dendritogenesis in melanocytes for feather pigmentation

**DOI:** 10.1038/s42003-024-06284-5

**Published:** 2024-05-17

**Authors:** Dong-Hwan Kim, Joonbum Lee, Jae-Kyun Ko, Kichoon Lee

**Affiliations:** 1 Department of Animal Sciences, The Ohio State University, Columbus, OH USA; 2 Department of Surgery, Davis Heart and Lung Research Institute, The Ohio State University, Columbus, OH USA; 3 Present Address: Pathology Advanced Translational Research Unit (PATRU), Department of Pathology & Laboratory Medicine, Emory University, Atlanta, GA USA

**Keywords:** Embryogenesis, Morphogenesis

## Abstract

Limited studies using animal models with a few natural mutations in melanophilin (Mlph) provided partial functions of Mlph in melanosome trafficking. To investigate cellular functions of Mlph, especially ZnF motif of Mlph, we analyzed all three Mlph knockout (KO) quail lines, one and two base pair (bp) deletions as models for total KO, and three bp deletion causing deletion of one Cysteine (C84del) in the ZnF motif. All quail lines had diluted feather pigmentation with impaired dendritogenesis and melanosome transport in melanocytes. In vitro studies revealed capability of binding of the ZnF motif to PIP3, and impairment of PI3P binding and mislocalization of MLPH proteins with ZnF motif mutations. The shortened melanocyte dendrites by the C84del mutation were rescued by introducing WT Mlph in vitro. These results revealed the diluted feather pigmentation by Mlph mutations resulted from congregation of melanosomes in the cell bodies with impairment of the dendritogenesis and the transport of melanosomes to the cell periphery.

## Abstract (web summary)

This study presents the essential functions of the ZnF motif in sorting of the melanophilin protein and novel functions of melanophilin in dendritogenesis in melanocytes.

## Introduction

Melanogenesis is a complex process through which melanin pigments in melanosomes are produced. Melanosomes containing pigment are synthesized in melanocytes and transferred to keratinocytes inducing pigmentation^1^. After initiation of melanin production, the pigmented melanosomes are transported along melanocyte dendrites, released from these dendrites and there is uptake by keratinocytes through cellular transport pathways^2^.

Melanophilin (Mlph) is a member of the Rab effector family, mainly consisting of three binding domains, Rab27 binding domain (R27BD) at the N-terminus, myosin-Va (MyoVa) binding domain (MBD), and actin-binding domain (ABD) at the C-terminus. As a linker, Mlph binds to two major proteins, the small GTPase Rab27a (Rab27a) and MyoVa to form a Mlph-Rab27a-MyoVa tripartite protein complex^3^. The tripartite protein complex involves transport of melanosomes from the terminal ends of microtubules and insertion of actin filaments in the periphery of melanocytes^4–6^. Functional loss of the three proteins in humans, MyoVa, Rab27a, and Mlph, leads to development of Griscelli syndrome (GS) type 1, 2, and 3, respectively^7^. Unlike humans with GS1 and GS2 that have common hypopigmentation and a neurologic dysfunction and humans with GS2 that have a unique immunologic disease, humans with GS3 have only hypopigmentation^7^. Hair and feather are structurally different. Major structural components of hair are medulla, cortex, and cuticle^8^. Feather is composed of rachis, barbs, and barbules^9^. However, similar to a symptom of GS2, there was diluted hair or feather colors caused by Mlph mutations in mice^10^, rabbits^11^, mink^12^, and chickens^13^.

The FYVE (present in Fab1p, YotB, Vac1p, and EEA1) is a domain composing of two Zn^2+^ ions with eight cysteines and is associated with vesicular membranes via binding to various phospholipids^14^. The FYVE domain proteins are involved in endosomal sorting/trafficking of vesicles containing various molecules via phosphatidylinositol 3-phosphate [PtdIns(3)P, also known as PI3P]^15,16^. Similar to the endosomal FYVE proteins, most of exophilin families have a zinc finger (ZnF) motif^17^, and Rabphilin-3A (exophilin1) and Mlph (exophilin3) have the FYVE-related domain which is structurally similar to FYVE. These exophilin proteins, however, lack the three signature sequences of the canonical FYVE domain: WxxD motif, R(R/K)HHCRxCG patch, and RVC motif^14^. Furthermore, Rabphilin-3A functions in cellular trafficking, but does not bind to PI3P^18^, indicating there are distinct functions of proteins containing FYVE-related domains from the FYVE domain proteins. Although results from numerous studies indicate there are functions of Mlph in melanosome transportation, functions of the FYVE-related domain in Mlph have not been extensively investigated, especially using in vitro and in vivo approaches.

Although animals with natural mutations in R27BD^10,13^ or MBD^12^ of Mlph were used to explain specific functions of each domain in melanosome transportation, complete loss of function in the *Mlph* gene by conducting genome editing procedures provide novel results for elucidating functions of the encoded protein. To elucidate the functions of Mlph in melanosome transportation and hypopigmentation, we previously produced genome-edited quail lines^19^: two knockout quail lines with one or two base pair (bp) deletions resulting in premature stop codons serving as the first animal models with complete loss-of-function, and one mutant quail line with three bp deletions resulting in deletion of one cysteine residue within the ZnF motif serving as a model with partial loss-of-function. More specifically, site-directed mutagenesis in the ZnF motif of the *Mlph* gene was performed for overexpression of various *Mlph* mutant genes in vitro to understand biochemical and cellular functions of the ZnF. In the current study, we identified novel functions of Mlph, as well as specific functions of the ZnF motif in regulation of melanosome transportation, and dendritogenesis in melanocytes.

## Results

### Mlph protein has a FYVE-related domain containing two zinc fingers

**Fig. 1:**
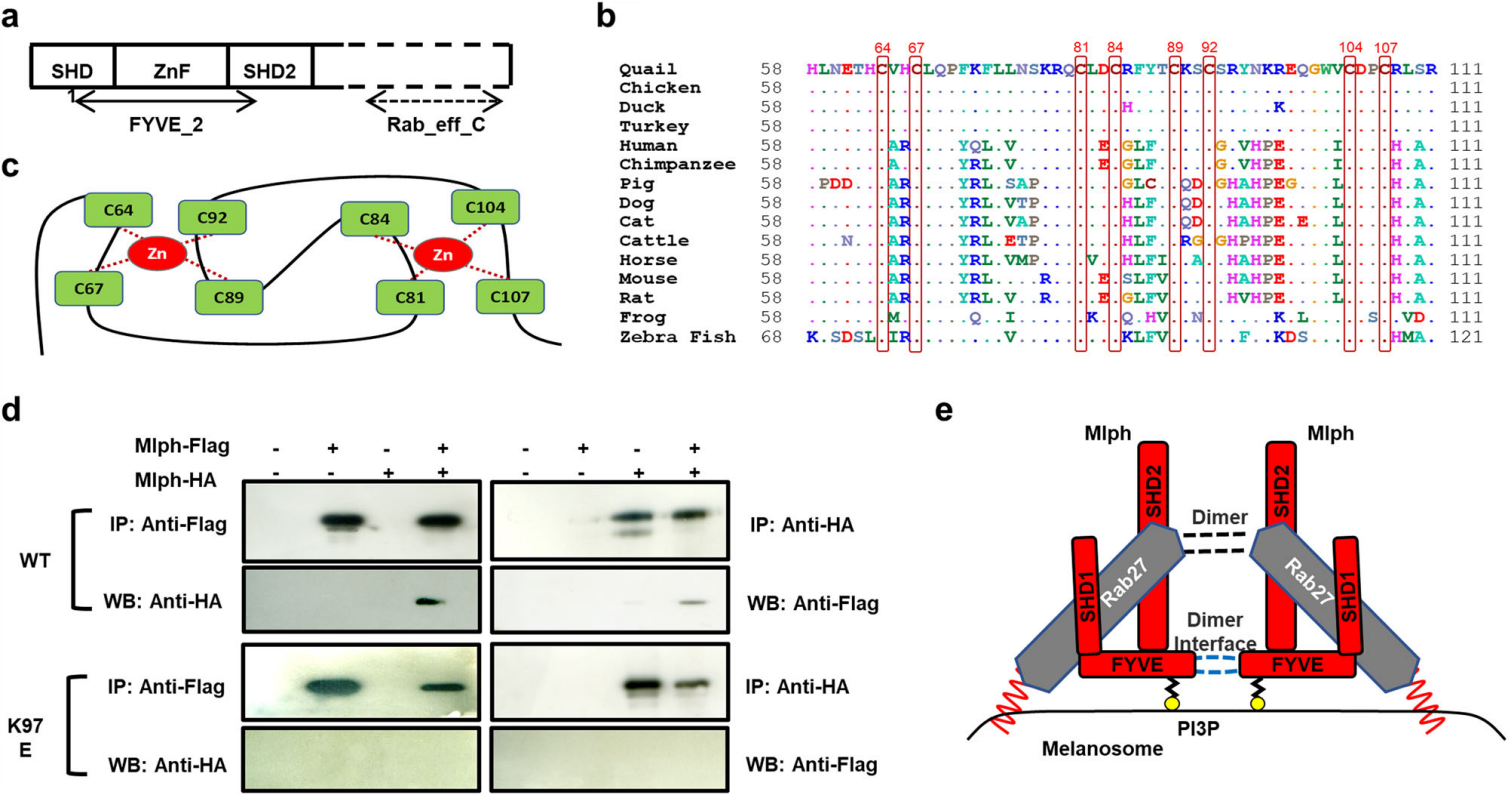
Zinc finger motif of Mlph binds to phosphatidylinositol 3-phosphate and forms homodimerization. **a** Schematic representation of Mlph. FYVE_2 motif is located at the N-terminus, and Rab_eff_C is at the C-terminus of Mlph protein. Synaptotagmin-like protein homology domain (SHD) 1 and SHD2 regions bind to Rab27a. Zinc finger (ZnF) is a cysteine-rich motif containing with two zinc ions. **b** Multiple alignments of the ZnF motif of Mlph in various animal species. All protein sequences were obtained from the database of the National Center for Biotechnology Information (NCBI, https://www.ncbi.nlm.nih.gov/): XP_015723580.1-Quail; NP_001108552.1-Chickens; XP_032045867.1-Ducks; XP_031409947.1-Turkeys; NP_001035932.1-Humans; XP_016806270.1-Chimpanzees; NP_001092064.1-Pigs; NP_001096689.2-Dogs; NP_001073123.1-Cats; NP_001075066.1-Cattle; XP_023498417.1-Horses; NP_443748.2-Mice; NP_001012135.1-Rats; NP_001120194.1-Frogs; XP_005167684.1-Zebra Fish. **c** A depiction representing the expected structure of the ZnF motif of Mlph. **d** Homodimeric interaction of Mlph. Several combinations of WT or mutant Mlph constructs were co-transfected into the 293FT cells. After 48 h of transfection, proteins were immunoprecipitated and immunoblotted to detect protein interactions. **e** Schematic illustration of Mlph-Rab27 complex in melanosome. Rab27 is anchored on the melanosome membrane through geranylgeranylation of Rab27 and homodimer of Rab27 binds to SDH1 and 2 of Mlph. Mlph can also homodimerize and bind to PI3P.

The information included in the NCBI database indicates that Quail Mlph contains two major domains, a FYVE-type zinc finger (pfam02318) in the N-terminus and Rab effector MyRIP/melanophilin (pfam04698) in the C-terminus of Mlph protein (Fig. 1a). The sequences of the ZnF motif for different animal species listed in Fig. 1b were obtained from the NCBI database. With multiple alignment of the sequences, it was ascertained that there is a conserved cysteine repeat motif [CX(2)CX(13)CX(2)CX(4)CX(2)CX(11)CX(2)C] (Fig. 1b). These eight cysteines are composed of two zinc fingers, with the underlined cysteines depicting the cysteine amino acid for the one zinc finger and the letters that are not underlined represent the cysteine amino acid for the other zinc finger. These structural configurations are a canonical FYVE finger stabilized by two Zn^2+^ surrounded by eight cysteines (Fig. 1c).

### The FYVE-related domain is required for binding of Mlph to phosphatidylinositol monophosphate

Considering FYVE domain binds to PI3P^20^, we investigated whether Mlph binds to PI3P and mutations in the FYVE-related domain of Mlph affect binding to PI3P. There was distinct binding of WT Mlph to phosphatidylinositol 3-phosphate and there was relatively less binding of WT Mlph to phosphatidylinositol 4- and 5-phosphate residues (Fig. S1). When there was substitution of cysteine residues at the 67th or 92nd to alanine in the first zinc finger, there was no detectable binding of Mlph to PI3P (Fig. S1). To evaluate the function of the second zinc finger, there was both deletion of cysteine at the 84th residue and substitution of cysteine at the 107th residue with alanine with the result being there was no detectable binding of Mlph to PI3P (Fig. S1). In addition, substitution of two amino acids (KR) in putative PI3P binding motif with two alanine residues (KR78-79AA) resulted in no detectable binding of Mlph to the phospholipid residues (Fig. S1). The similar band densities of the Flag-tagged Mlph proteins, when there was conducting the Western blot analysis (Fig. S2), indicated there was a similar quantity of Mlph proteins that were used for making evaluations using the phospholipid-binding assay, further indicating the validity of this assay for estimating quantities of Mlph protein.

### Mlph is dimerized through FYVE-related domain

Endosomal proteins containing the FYVE domain are generally dimerized via the FYVE domain^21^. Interestingly, the structural motif of the FYVE-related domain in the Mlph protein is very similar to these endosomal proteins, leading us to predict potential dimerization of Mlph protein via the FYVE-related domain between the 92nd to 104th amino acids. To test this hypothesis, we generated Flag or HA-tagged WT and K97E Mlph proteins. Results from co-immunoprecipitation of the two proteins indicated there was an interaction between Mlph WT-Flag and -HA proteins (Fig. 1d). Whereas, when the 97th amino acid of Mlph was substituted from lysine to glutamic acid (K97E), the mutant Mlph did not have the capacity for inducing dimerization (Fig. 1d). These results suggest that Mlph has dimer interface in the FYVE-related domain.

### Mlph co-localizes in the F-actin network at cell periphery and the FYVE-related domain disruption leads to an altered Mlph location

**Fig. 2:**
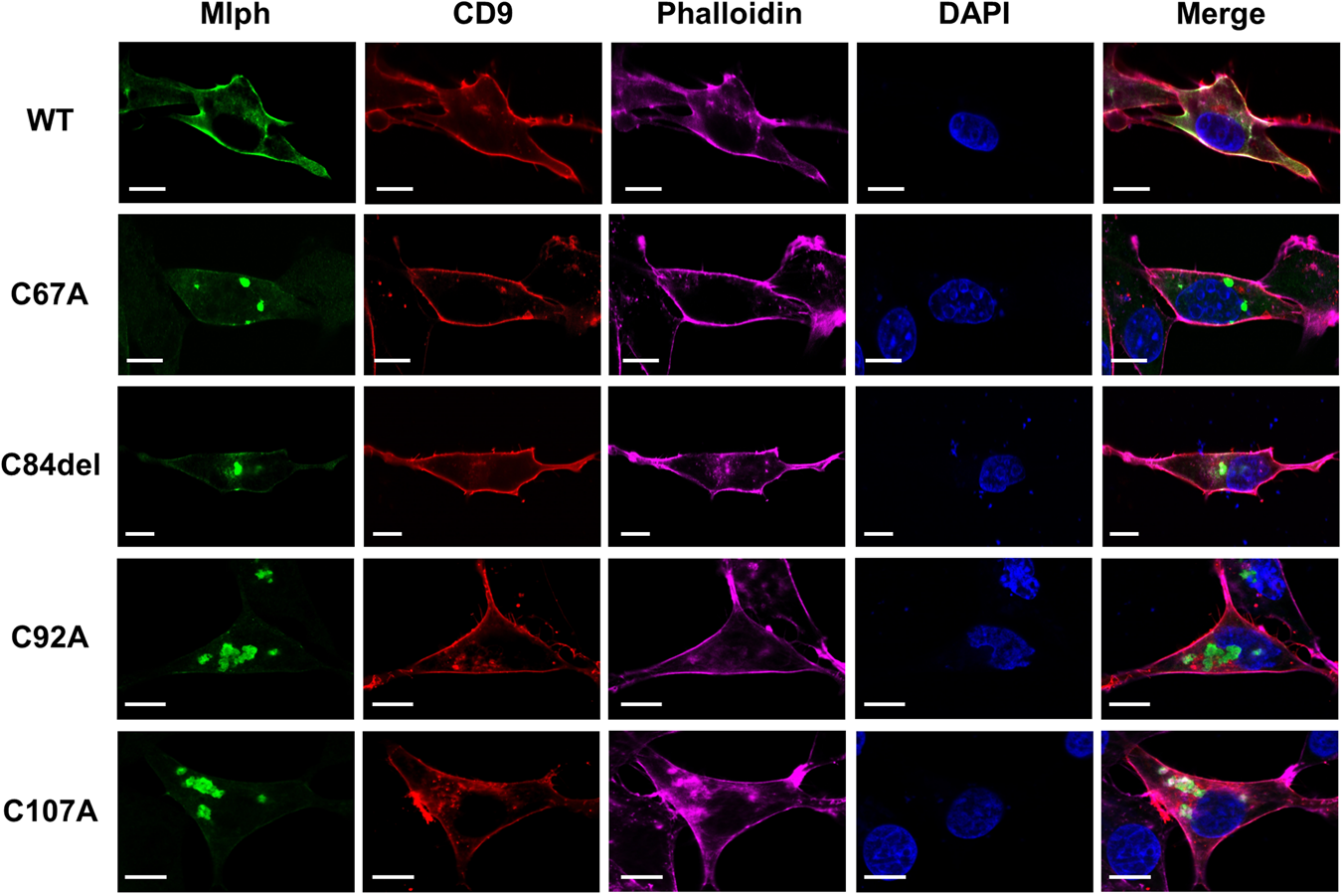
The ZnF motif is essential for proper localization of Mlph in the cell periphery. Flag-tagged Mlph WT or mutants, and pmCherry-CD9 constructs were co-transfected into QM7 cells. After 48 h of transfection, cells were fixed and stained, and subsequently visualized using a confocal microscope. pmCherry-CD9 was used for staining the cell periphery, and phalloidin for actin. DAPI was used for nuclei staining. Scale bar: 10 μm.

The Mlph protein is mainly localized on peripheral melanosomes in melanocytes^22^ and is involved in transport of the melanosome from melanocytes to the keratinocytes^23^. Most exophilin proteins containing the ZnF motif are involved in the trafficking of exosomes^17^. The CD9 protein modulates exosome transportation, and is normally localized in the cellular periphery where there is an abundance of F-actin^24^. Both CD9 and F-actin, therefore, serve as cellular peripheral markers. Because Mlph also contains the ZnF motif in the FYVE-related domain at the N-terminus, and also the F-actin-binding domain at the C-terminus, we investigated whether WT Mlph co-localizes with both CD9 and F-actin at the periphery of cells. There was also evaluation of whether mutations in the FYVE-related domain of Mlph has effects on cellular localization of the mutant Mlph proteins. The WT Mlph co-localized with CD9 and phalloidin, indicating peripheral localization of Mlph protein. Interestingly, however, mutations of cysteine residues (C67A, C84del C92A, and C107A) in the FYVE-related domain disrupted normal localization in cellular periphery but there was granulation inside of the QM7 cells (Fig. 2). After further investigation of the localization of the Mlph proteins, it was determined that the mutant Mlph proteins co-localized primarily with either the endosomal marker (FYVE) or lysosome (Fig. S3).

### Both knockout of Mlph and deletion of a cysteine in the ZnF of Mlph resulted in feather color dilution in quail

**Fig. 3:**
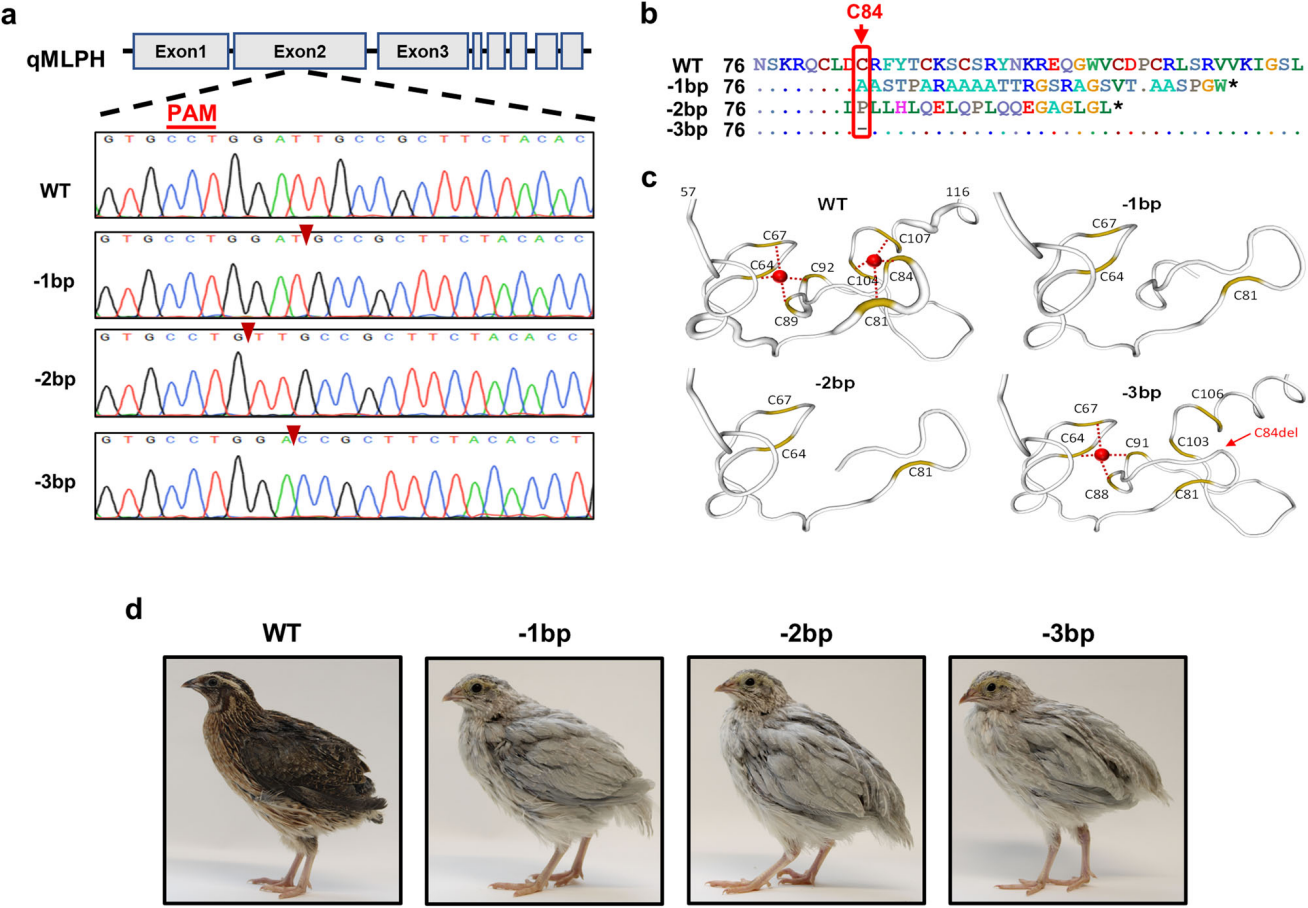
Description of three different quail lines with genome edition in Mlph gene. **a**, **b** Targeting loci of quail Mlph and sequencing analysis of mutants. To target the ZnF motif of Mlph in quail, exon 2 was targeted and results from sequencing analysis indicated 1, 2, or 3 base pair (bp) deletions of Mlph gene (−1, −2, or −3 bp, respectively) resulting in mutations around the 84th amino acid. **c** Comparisons of the predicted three-dimensional structures of ZnF motif in WT quail Mlph with three different mutants. Red circle represents Zn2+ ion. **d** Phenotypic comparisons of Mlph WT with the mutant quail lines. WT quail have dark brown plumage, whereas quail of all mutant lines have silver-gray plumage.

Several lines of Japanese quail with mutations in the *Mlph* gene were previously produced by microinjection of an adenoviral CRISPR/Cas9 vector into quail blastoderm^19^. The guide RNA (gRNA) was designed to target the ZnF motif in exon 2 of the *Mlph* gene, resulting in several mutant quail lines. Among the mutant quail lines, there were two representative quail lines with a 1 and 2 bp deletion (−1 and −2 bp, respectively) selected to conduct gene knockout studies because these mutations induce a frame shift and subsequently there is a premature stop codon for protein synthesis (Fig. 3a, b). Interestingly, the 3 bp deletion (−3 bp) in one mutant quail line results in deletion of the 84th residue (cysteine) in the ZnF motif (Fig. 3a, b). There was a specific interest in investigating effects of deletion of 84^th^ residue (cysteine) in the ZnF motif on functions of Mlph in vivo because induced mutations at the site of cysteine residues and deletion at the 84th cysteine within the ZnF motif resulted in the loss of capacity to bind PI3P and mislocalization of the mutant proteins in vitro.

Because eight cysteine residues within endosomal FYVE domain are essential for structural integrity of ZnF motif^14^, there was use of the SWISS-MODEL Repository ^25^ to predict structural changes of the FYVE-related domain that resulted from the mutations (−1, −2, and −3 bp) compared to the WT Mlph protein. As depicted in Fig. 3c, the WT Mlph contains two Zn^2+^ ions surrounded by eight cysteines forming two ZnFs, but in the mutant quail (−1 and −2 bp) there was disruption of both ZnFs and in the individuals with the −3 bp mutation there was only the initial ZnF. Disruption of the ZnF motif in individuals of all three mutant quail lines induced an obvious phenotypic difference with there being silver-gray plumage on the whole-body compared to the WT quail with only dark brown plumage (Fig. 3d).

### The Mlph mutation impaired melanin pigmentation dispersion in embryonic feathers, resulting in silver-gray downy feathers

**Fig. 4:**
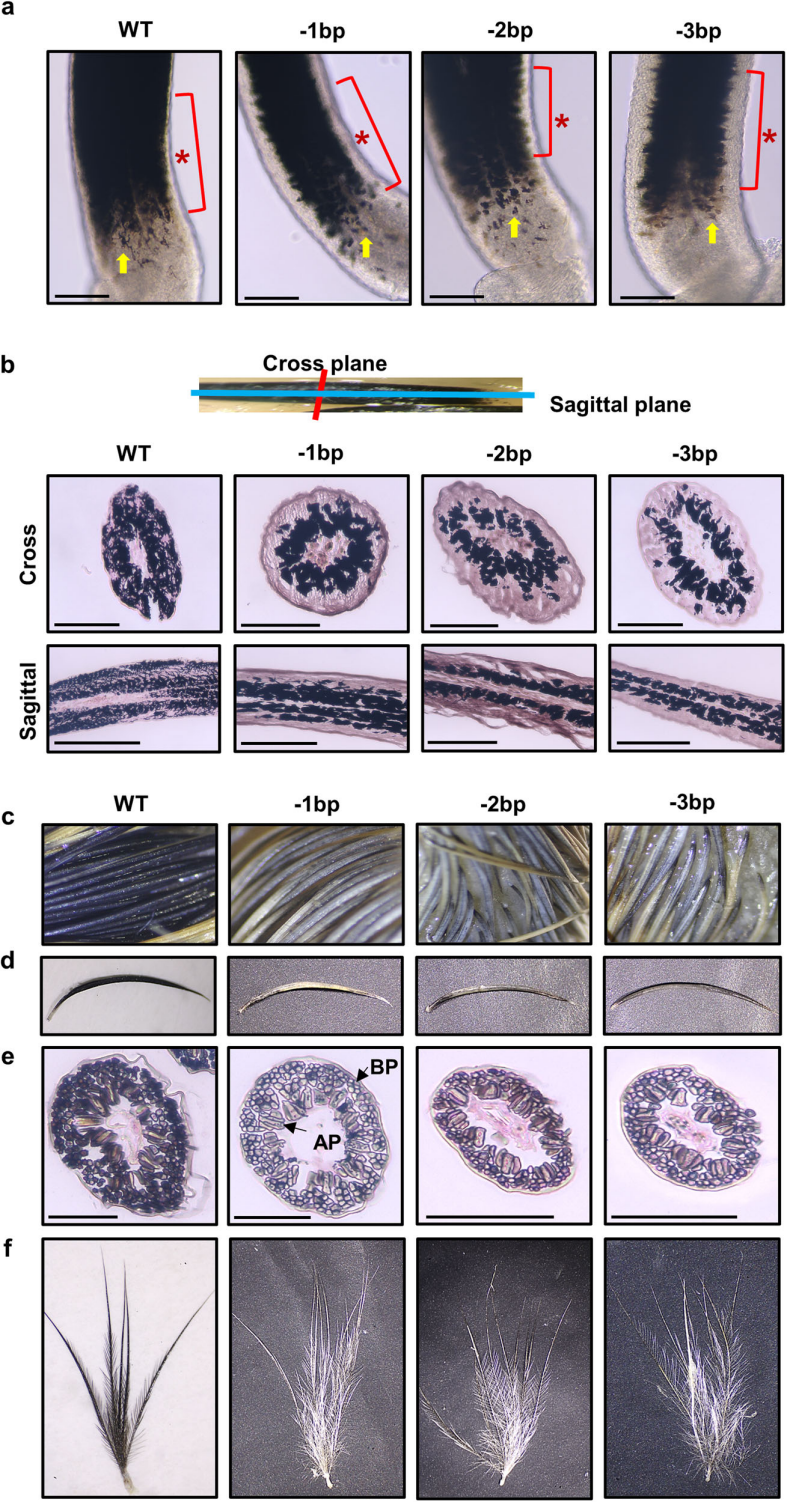
Changes of feather pigmentation resulting from Mlph mutations. **a** Comparisons of feather pigmentation at base of developing embryonic feathers at 10 days after initiation of egg incubations (E10). Asterisk indicates dispersion of melanin pigmentation during longitudinal growth of feather buds and the arrow depicts dispersion of melanin pigmentation in melanocytes near the base of embryonic feather follicles. **b** Pigmentation patterns in embryonic feather bud formation at E10 stage. Embryonic feathers were sectioned in cross or sagittal planes, and then Fontana-Masson stain was applied for identifying melanin dispersion in the feathers. **c**–**f** Different patterns of feather pigmentation among the quail of different lines at the E15 stage. Among dorsal feathers (**c**), one representative feather was removed (**d**), and cross-sectioned for Fontana-Masson staining (**e**). AP axial plate, BP barbule plate. One dorsal feather was dried and the feather sheath was removed to visualize color differences (**f**). All scale bars are indicative of 100 μm.

Although melanin pigmentation dynamically occurs during feather bud formation in the embryos, spatiotemporal dispersion of melanin pigmentation in developing feather buds has not been described in detail. Considering the appearance of feather buds at E9 in quail^26^, we investigated effects of Mlph mutation on regulation of pigmentation in developing embryonic feathers at the E10 and E15 stages. Stereoscopic views near the base of intact feather buds at the E10 stage, there was black pigmentation in melanocytes detected along the stretched dendrites in the individuals of the WT; whereas, mutant birds tended to have pigmentation in the cell bodies (arrow in Fig. 4a). During the longitudinal growth of WT feather buds, the peripheral area of the base of intact feather buds is gradually filled with the black pigmentation. In contrast, black pigmentation does not spread toward the peripheral layer of the feather buds of mutant birds, resulting in there being a transparent peripheral layer of the entire feather bud (asterisk in Fig. 4a).

Consistent with the results from longitudinal views (Fig. 4a), in the cross sections of embryonic feather buds there was distinct black pigmentation that was widely dispersed from the inner to peripheral layer of the feather buds in WT quail. The pigmentation, however, was restricted in the inner layer of the feather buds in the birds of the Mlph mutant quail lines (−1, −2, and −3 bp; Fig. 4b). In addition, in the sagittal sections of the embryonic feathers there was definitive pigmentation both in the inner and peripheral area of the feather buds in the birds of the WT birds but only in inner area in birds of all mutant lines (Fig. 4b). Furthermore, in both cross and sagittal sections, there are pigmentations along with long well-developed dendrites in the feather buds of WT birds but restricted pigmentation in cell bodies in birds of all mutant lines (Fig. 4b).

Colors of the dorsal feathers at E15 stage were black and brown in birds of the WT but silver-gray and light brown in those of the mutant lines (Fig. 4c). In cross sections of the representative prenatal down (Fig. 4d) at the E15 stage there was black pigmentation that was evident in the axial plate (AP) and barbule plate (BP) in the birds of WT line (Fig. 4e), resulting in black feather pigmentation. In the cross section, however, there was a lack of black pigmentation in AP and BP in feathers of birds of mutant lines (Fig. 4e). To further evaluate these differences with the visual evidence of actual color of embryonic feathers, one strand of prenatal down from WT or mutant quail embryos (Fig. 4d) was dried and the feather sheath was removed, allowing for unfolding and opening of the barb and barbules. Colors of the barb and barbules were black in birds of the WT but silver-gray in birds of the mutant lines (Fig. 4f).

### Mlph is required for normal dendritogenesis of melanocytes

**Fig. 5:**
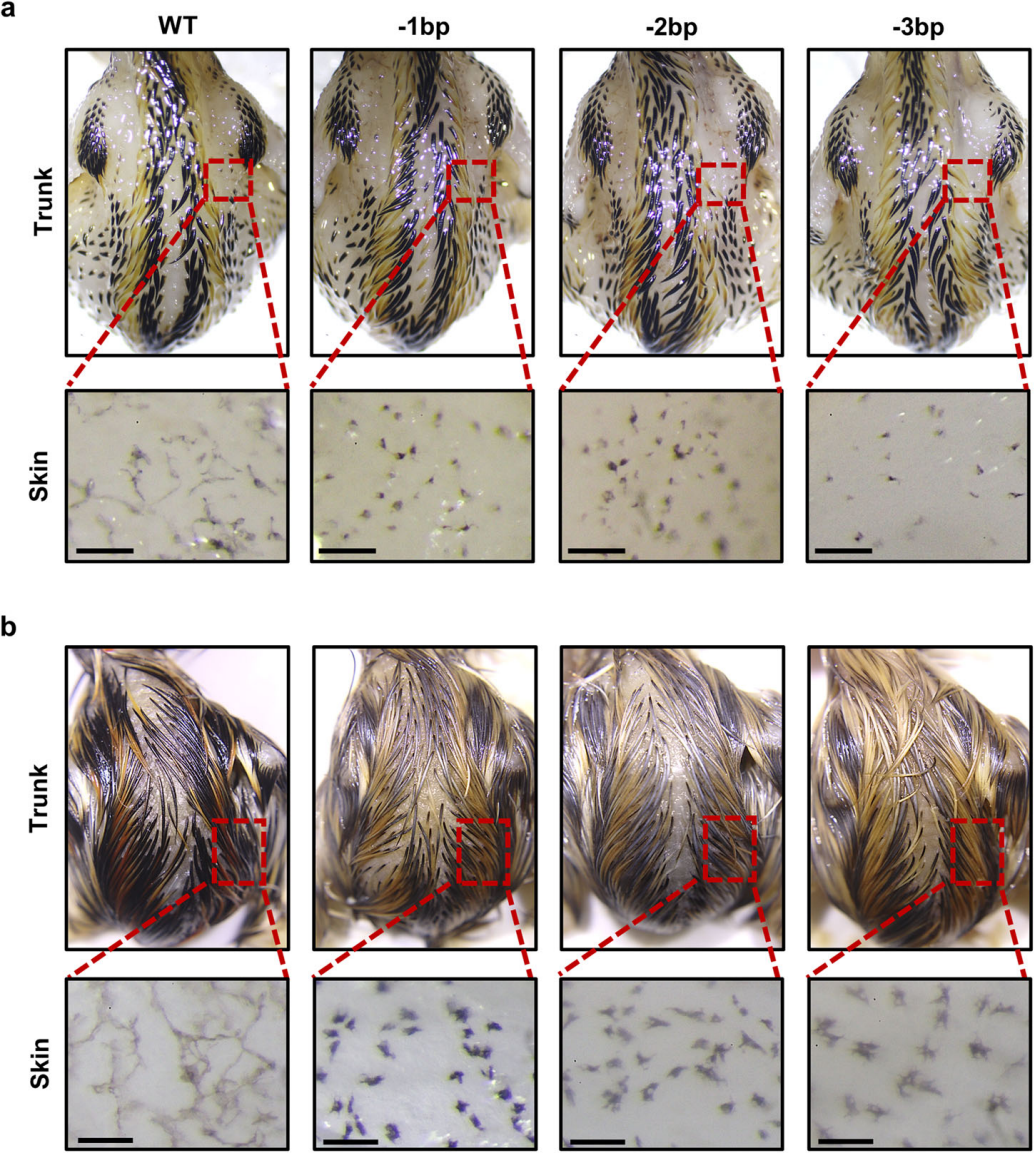
Stereomicroscopic visualization of feathers and dorsal skin areas of Mlph mutant birds at the two embryonic stages. Dorsal visualization of trunks and skin of birds of Mlph WT and gene-edited lines at the E10 (**a**) or E15 (**b**) stages. Scale bar: 100 μm.

Because Mlph mutations may potentially affect melanocyte dendricity in embryonic feathers (Fig. 4), there was initial investigation of whether dendricity of melanocytes in the skin was modulated in birds with Mlph mutations. At the E10 stage, in the stereoscopic views of dorsal trunks, there were similar patterns of multiple longitudinal stripes of black and brown feathers in birds of the different groups (WT, as well as −1, −2, and −3 bp; trunk in Fig. 5a). There were similar patterns of these strips of feathers detected in birds of all lines although those of the three mutant lines had diluted feather pigmentation at the E15 stage, with there being pigmentations of a black to silver-gray and brown to yellowish color (trunk in Fig. 5b). Stereoscopic images of surface of dorsal skins were evaluated to investigate the morphology of melanocytes in the skin of the birds of the WT and Mlph mutant lines at the two embryonic stages. At the E10 stage, melanocytes of WT birds had stretched dendrites of a dark color; whereas skin melanocytes in the birds of the Mlph mutant lines (−1, −2, and −3 bp) tended to have dark colored cell bodies compared to those of the WT birds (skin in Fig. 5a). At the E15 stage, these characteristics of melanocytes were more distinct between the birds of the WT and mutant lines (skin in Fig. 5b).

**Fig. 6:**
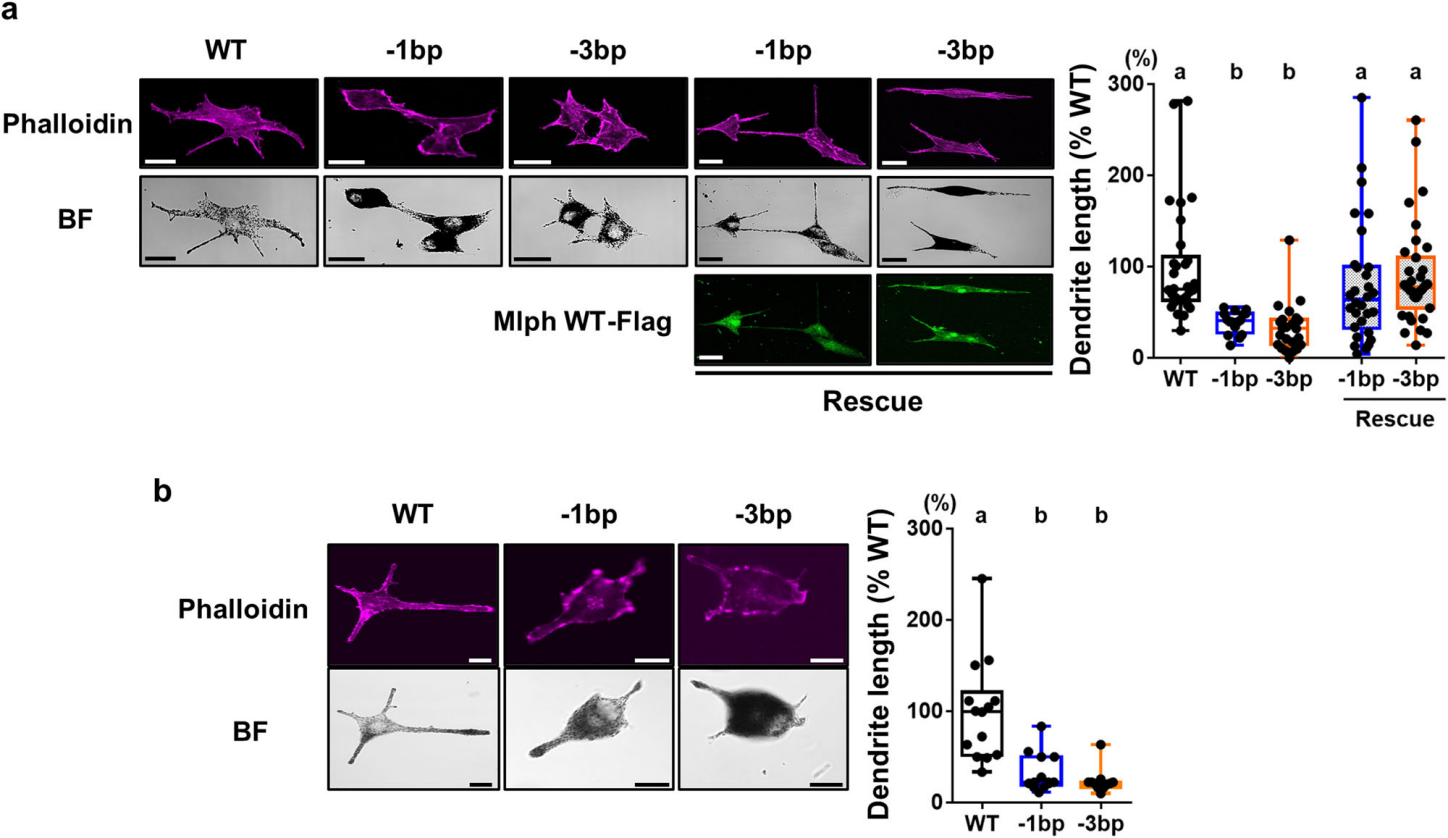
Effect of Mlph mutations on dendritogenesis in primary melanocytes. **a** The dendrite length of the primary melanocytes isolated from skin of WT and gene-edited Mlph quail lines (WT, −1 or −3 bp) and evaluated 3 days after seeding using phalloidin staining procedures. To investigate whether the gene-edited alterations of dendrite length overcome by WT Mlph presence, Mlph mutant melanocytes isolated from the −1 or −3 bp quail lines were cultured, and, 1 day after seeding, a construct expressing Flag-tagged Mlph WT was transfected into the cells. At 2 days after the transfection, cells were fixed and stained using anti-Flag and Phalloidin. To analyze dendricity, dendrite lengths were determined by evaluating cells: 30 for WT, 16 for −1, and 23 for −3 bp; for evaluations when there was WT Mlph present, 30 for −1 bp, and 35 for −3 bp. Percentages for dendrite length are presented relative to the WT as means ± SEM. Scale bar: 20 μm. **b** Cells including melanocytes were isolated from embryonic feathers of WT and gene-edited Mlph quail lines at E9, and cultured for 3 days to measure dendrite length using phalloidin staining. To analyze dendricity, dendrite lengths were determined by evaluating cells: 14 for WT, 13 for −1, and 11 for −3 bp. Scale bar: 10 μm. In the A and B, percentages for dendrite length are presented relative to the WT as means ± SEM. Multiple means were compared using a one-way ANOVA utilizing GraphPad Prism software, version 6.02. There were considered to be mean differences when there was a *P* < 0.05.

In general, dendricity of normal skin melanocytes can be determined by quantifying the dark color of stretched dendrite of melanocytes because dark color melanosomes are normally transported to the end of dendrites^27^. With this method, however, there may not be accurate quantification of the in vivo dendricity of birds of mutant lines because length of area with dark color may not represent length of dendricity due to possible changes in magnitude of melanosome transport along dendrites and/or dendritogenesis in birds where there were Mlph mutations. To further investigate Mlph functions in melanocyte dendricity, there were evaluations of dendricity of the embryonic skin melanocytes in culture where cells were stained with phalloidin to quantify dendrite lengths. These lengths were markedly less in cells from birds with Mlph mutations (−1 and −3 bp, 39% ± 3 and 33% ± 6, respectively) compared to the WT birds (100% ± 11) (Fig. 6a). Similarly, shorter dendrite lengths were also exhibited in embryonic (E9) feather melanocytes in cultures (100% ± 15 for WT, 32 ± 6 for −1 bp, and 24% ± 4 for −3 bp, Fig. 6b). Furthermore, there was determination of whether the short dendricity in the primary melanocytes of birds with Mlph mutations can be rescued by overexpression of WT Mlph gene. Findings depicted in Fig. 6 indicate the melanocyte dendricity of the birds of mutant lines was similar to that of WT birds when there was overexpression of the WT Mlph gene (−1 and −3 bp, 80% ± 12 and 90% ± 9, respectively; Fig. 6a). Disruption of the *Mlph* gene, therefore, resulted in impairment of melanocyte dendritogenesis which can be overcome by expression of the WT Mlph gene, clearly indicating the important functions of Mlph in melanocyte dendricity.

## Discussion

Feather pigmentation in avian species is controlled by two primary cells, melanocytes producing melanin pigments in melanosomes, and keratinocytes receiving the pigmentation from melanocytes. The Mlph, Rab27a, and MyoVa proteins modulate the processes of melanosome transportation as a tripartite protein complex. In the current study, we investigated functions of Mlph in regulation of pigmentation using in vitro and in vivo approaches including genome-edited quail. The findings of the Mlph functions in this study were: (I) Mlph is associated with melanosome through binding of the ZnF motif to PI3P and disruption of the ZnF inhibits PI3P binding, (II) Mlph localizes in the cell periphery, and this migration is inhibited when there are mutations disrupting cysteine residues in the ZnF motif, (III) there is abnormal localization of the Mlph protein when there is ZnF mutation leading to a lack of capacity to transport melanosomes during feather development, resulting in diluted feather pigmentation, and (IV) Mlph modulates dendritogenesis of melanocytes. In addition, quail with a total knockout of the *Mlph* gene were produced and used for the first time rather than animals with natural mutations in various domains, resulting in identification of additional novel functions of Mlph in melanocytes. Furthermore, deletion of the 84th cysteine in ZnF motif in the Mlph mutant line results in abnormal cellular localization of Mlph, and leads to the similar silver-gray phenotype in feathers resulting from the complete loss of *Mlph* gene in the two knockout quail lines, indicating the importance of proper localization of Mlph for regulation of melanosome transportation.

The PI3P is mainly located in membranes of endosomes^14^ and the FYVE finger is a cysteine-rich double zinc-binding domain which binds to PI3P^28^. Most of the PI3P is associated with cellular pathways (e.g., endocytosis, exocytosis, and autophagy) by spatiotemporal control of membrane trafficking ^29^. Furthermore, similar to the endosomal proteins containing FYVE, most exophilin proteins have the double zinc domain including Mlph^17^. Results in the present study are indicative of the importance of ZnF motif in Mlph for binding to PI3P because mutations of the ZnF motif hinder interaction of Mlph with PI3P. The EEA1 and Hrs proteins containing the FYVE domain are also homodimerized^20,21^. Because Mlph contains FYVE-related domain, there was investigation in the present study of whether Mlph can be homodimerized. It was clear in the present study that Mlph is in a homodimeric complex form and mutations in the ZnF motif inhibit the dimerization, indicating the intact ZnF motif requires Mlph dimerization.

The Mlph was detected on the periphery of QM7 cells in the present study, however, mutations in the ZnF motif of Mlph disrupted localization of the protein in the cells with there being detection in both endosomes and lysosomes. Furthermore, primary melanocytes isolated from embryonic skin of the −1 bp quail line that are devoid of endogenous expression of Mlph, were used to evaluate localization of exogenous mutant Mlph proteins. The results from cellular localization evaluations further indicated that Mlph (WT) is mainly located at the periphery of melanocytes isolated from skins of the −1 bp quail line, but mutant Mlph (C84del) was not localized at the periphery of these cells (Fig. S4). These results are indicative that disruption in cellular localization of Mlph in birds of the −3 bp mutant line leads to a lack of melanosome transport, possibly resulting in cessation of migration of the mutant proteins at the endosome stage or lysosomal degradation of proteins from birds of the −3 bp mutant line.

Feather growth depends on the proliferation of collar keratinocytes and barb cells, and there are darker feather colors with accumulation of melanin which is synthesized in melanocytes. After production of melanin, it is transferred to keratinocytes^9^. During longitudinal growth of WT embryonic feather buds, we report for the first time that there is gradual filling of melanin pigmentation at the base of feather buds indicating there is spatiotemporal transportation of melanin pigments into peripheral layers (keratin layers). However, in the present study, impairment of transferring melanin pigments to the keratin layers of developing embryonic feathers by genome-editing of Mlph could be associated with less dendritic stretching in feather follicles. In addition, findings when there were histological assessments of feathers at the E10 stage, further confirmed that there was widely dispersed black pigmentation in the WT quail but there was not this transport into these outer layers of the feathers in the genome-edited quail. Results were definitive when there was further analysis of feathers at the E15 stage because unlike the pigmentation of WT birds there was not pigmentation present in the region of the barbule and axial plates of feathers in quail of the mutant lines. The results from the genome-edited quail lines were also definitive because it was ascertained that there were essential functions of Mlph in spatiotemporal processes in feather pigmentation during embryonic feather development.

The Mlph protein might directly bind to melanosomes^1^ and several proteins containing the FYVE domain have a membrane insertion loop motif ^30^. Based on results from the present and previous studies, interactions of Mlph and Rab27 on melanosomes were predicted in Fig. 1e. There are depictions of homodimerized Rab27^31^ binding to two α-helix regions [synaptotagmin-like protein homology domain (SHD)1 and SHD2] at the N-terminus of Mlph^17,32^ of which mutation resulted in loss of Mlph binding to Rab27a^3,33^. The Mlph protein might be directly associated with melanosome through the PI3P binding motif in the FYVE-related domain with a homodimeric complex. In mice^10^ and chickens^13^, natural mutations in the SHD1 domain of Mlph led to a lack of binding to Rab27a and caused changes in hair and feather pigmentation, respectively. These findings are indicative that binding capacity of the SHD domain to Rab27a is essential for normal pigmentation. Although the ZnF motif of Mlph is located between two SHD domains, mutations of each cysteine residue (Fig. S5) or deletion of all eight cysteine residues^32^ in the ZnF motif did not affect the binding capacity of mutant Mlph proteins to Rab27a. Although the deletion of one cysteine residue (C84del) can bind to Rab27a, the silver-gray feather coloration by the C84del supports the essential functions of the ZnF motif in regulation of feather coloration that was not related to the Rab27a protein.

Results from previous studies indicate natural mutations in the R27BD and MBD of Mlph resulted in defects of melanosome transport with diluted hair or feather color in humans^7^, mice^10^, chickens^13^, and American mink^12^. In the current study, the gRNA was designed to induce mutations within the conserved cysteine repeat motif. Among the three mutant quail lines, birds of the two lines with deletion of 1 or 2 bp (−1 or −2 bp, respectively) had silver-gray colored feathers as a result of premature stop codons that resulted in deletion of half of the FYVE-related domain. The birds of the third mutant line with deletion of 3 bp (−3 bp) had a deletion of a single cysteine residue (C84del) within ZnF motif of Mlph protein, but all other domains remained intact. Interestingly, the −3 bp quail line also had silver-gray feather coloring similar to the birds of the two knockout lines (−1 and −2 bp). The in vitro findings in the present study are interpreted to validate that the ZnF motif is an essential domain for binding to PI3P and transport of Mlph to periphery of cells because the various mutations, including C84del, led to impairment in these functions of the ZnF motif resulting in the silver-gray feather color in the −3 bp quail line. The Mlph protein normally co-localizes with the peripheral actin cytoskeleton of melanocytes in which Mlph facilitates melanosome transportation from microtubules to actin filaments at the ends of dendrites for transport into keratinocytes^34^. In the current study, disruption of peripheral localization of Mlph by cysteine mutations in the FYVE-related domain in primary melanocytes led to induction of melanosome accumulation around peri-nuclei regions, resulting in impairment of melanosome transportation. Most of endosomal proteins containing the FYVE domain are initially sorted to the membrane of early endosomes that fuse to multivesicular endosomes and the proteins are subsequently associated with the membrane of final target vesicles for sorting and trafficking^15,16^. Mutations in the FYVE domain proteins including FENS-1^35^, SARA^36^, EEA1^37^, and Endofin^38^ can disrupt proper cellular localization to the early endosome, leading to impairment in transportation of target vesicles within cells. This is similar to what occurs when there is disruption of the FYVE domains that leads to disruption of the localization of protein and consequently impairment in trafficking of target vesicles. Results from the present in vitro and in vivo studies are the first evidence that among the exophilin family members involved in intracellular trafficking, with there being compelling evidence the ZnF motif in Mlph is essential for localization of Mlph protein for proper function in melanosome transportation and feather pigmentation.

The dendrite formation of melanocytes is an essential process for transport of mature melanosomes along the dendritic microtubules to the terminal ends where melanosomes are transferred to keratinocytes^39^. In the present study, an unexpected common phenotype in birds of all three lines is shorter than typical dendrites in melanocytes as determined by evaluations of the embryonic skin and at the base of feather buds. Because Mlph functions in combination with Rab27a or MyoVa, mutations of these co-functioning proteins were expected to result in a similar phenotype of short dendrites in melanocytes. Results from previous studies^5,40^ indicated mutations in Rab27a or MyoVa resulted in normal length of dendrites but impairment of melanosome transportation. Results from the present study indicate Mlph is involved in dendrite formation independent of functions in combination with Rab27a and MyoVa.

Numerous Rab proteins regulate dendritogenesis, and disruption of the Rabs can lead to impairment of dendrite outgrowth^41,42^. In melanocytes, both Rab27a and b, that are 72% homogenous in amino acid sequences^43^, bind to Mlph, and regulate melanosome transportation^44,45^. The Rab27a protein is a major factor, rather than Rab27b, in regulating transportation of melanosomes in melanocytes as indicated by impairment of melanosome transportation in Rab27a-deficient melanocytes in which there is Rab27b present. Interestingly, overexpression of *Rab27b* gene can result in overcoming of the impaired transportation of melanosomes in Rab27a-deficient melanocytes^44^, indicating there are similar functions of Rab27a and b in regulation of melanosome transportation. Considering the findings that the mutation in Rab27a in *ash* mice resulted in impaired melanosome transportation but normal dendritogenesis^40^ and regulation of melanosome transportation as induced by the Mlph/Rab27a axis, it was somewhat unexpected that disruption of Mlph affected dendritogenesis in melanocytes in the present study. A dominant negative Rab27b mutation inhibited formation and maintenance of melanocyte dendrites^46^, however, and this was similar to the impaired dendritogenesis in the absence of Mlph or C84del mutation in the ZnF in the current study. These findings in the present study suggest the Mlph/Rab27b axis could be a potential regulatory complex for dendritogenesis in melanocytes.

**Fig. 7:**
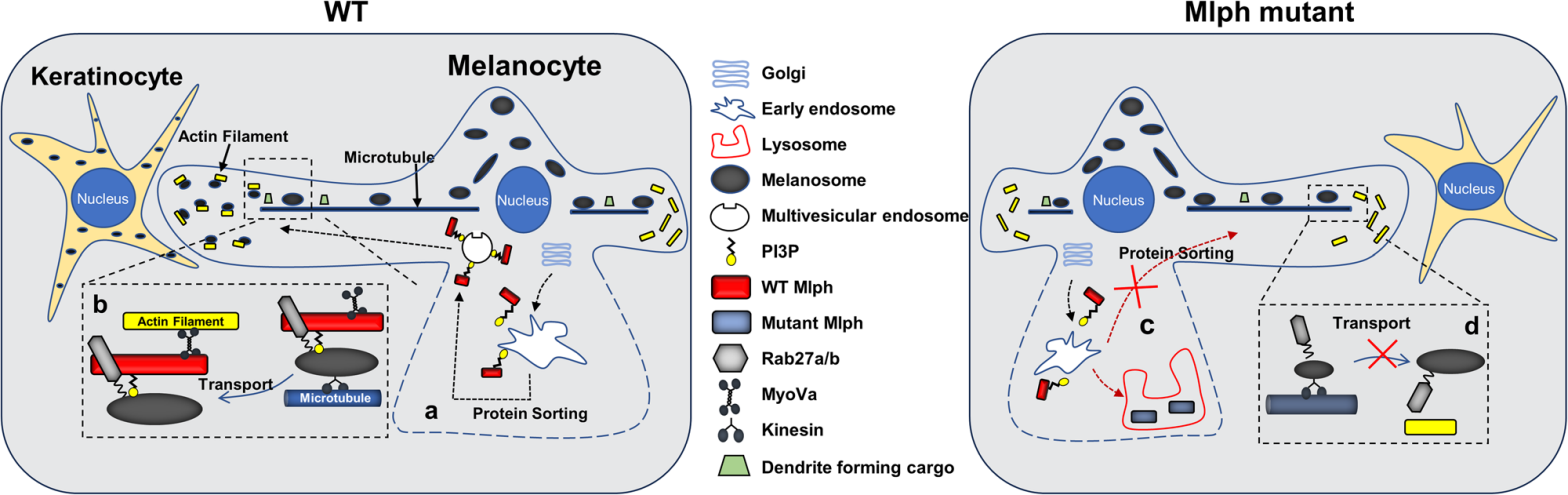
Graphical summary depicting functions of Mlph in regulation of dendritogenesis and melanosome transportation in melanocytes. **a** The WT Mlph protein might be transported via multivesicular endosomes to the periphery of the cells. **b** After Mlph is transported to the cell periphery, it facilitates transferring of melanosome from the microtubules to actin filaments. **c** Mutations in the ZnF motif of Mlph disrupt proper sorting of the Mlph protein, consequently resulting in aggregation in the lysosome and possible degradation of mutant Mlph. **d** The absence of Mlph at the cellular periphery impairs transportation of the potential vesicles containing dendrite forming substances at the proximal tips of growing dendrites, resulting in impaired dendritogenesis in melanocytes and congregation of melanosomes in the cell bodies.

Taken together, we defined functions of Mlph in the melanosome transport and cellular morphogenesis as illustrating in the Fig. 7. To reach the site of the Mlph action, synthesized Mlph protein is transported from the Golgi apparatus and sorted to endosomal vesicles. Subsequently, the WT Mlph protein might be transported via multivesicular endosomes to the periphery of the cells (Fig. 7a). After Mlph is transported to the cell periphery, it facilitates transferring of melanosome from the microtubules to actin filaments. Similarly, transport of potential vesicles containing building blocks for dendrite formation might be facilitated by Mlph, leading to dendrite extension, thus enabling transportation of melanosomes into keratinocytes at the proximal cellular location (Fig. 7b). Mutations in the ZnF motif of Mlph disrupts proper sorting of the Mlph protein, consequently resulting in aggregation in the lysosome and possible degradation of mutant Mlph (Fig. 7c). Due to disruption in cellular localization of Mlph, the absence of Mlph at the cellular periphery impairs transportation of the potential vesicles containing dendrite forming substances at the proximal tips of growing dendrites, resulting in impaired dendritogenesis in melanocytes and congregation of melanosomes in the cell bodies (Fig. 7d). Further research is needed for precise elucidation of functions of the Mlph ZnF motif in melanocytes. However, results from the present study are novel and definitive that the ZnF motif of Mlph has essential functions in cellular sorting/trafficking of Mlph protein, and Mlph regulates dendritogenesis in melanocytes.

## Methods

### Structure of ZnF motif and sequence comparisons

Based on a previous study^47^, domains of quail Mlph at the N-terminus were predicted and the sequences of ZnF motif in Mlph proteins in different species were compared using Clustal Omega (available at https://www.ebi.ac.uk/Tools/msa/clustalo/). The protein sequences were obtained from the database of the National Center for Biotechnology Information (NCBI, https://www.ncbi.nlm.nih.gov/). In addition, the structure of the ZnF motif in Mlph was predicted based on results from previously conducted studies^48^.

### Molecular cloning of WT and mutant Mlphs, Rab27a, and CD9 cDNAs

The cDNA encoding a full open reading frame of quail *Mlph* (XM_015868099.2), *CD9* (XM_015871499.2), and *Rab27a* (XM_015872903.1) was amplified from quail kidney cDNA by reverse transcriptase PCR using specific primer sets. For constructing expression vectors containing HA- or Flag-tagged *Mlph* and *Rab27a*, the PCR products were inserted into pCR2.1-TOPO (#450641, Invitrogen, Carlsbad, CA, USA) and cut with a restriction enzyme, EcoRI (#R0101, New England BioLabs, Ipswich, MA, USA). Each of the fragments was ligated into the pcDNA3.1 vector (#V79020, Invitrogen). Using the pcDNA3.1-qMlph-WT-Flag, vectors containing the various Mlph mutant genes (C67A, KR78-79AA, C92A, K97E, and C107A) were produced by conducting site-directed mutagenesis using procedures we previously published^49^. To construct overexpression vectors containing qMlph-1bp-Flag or -C84del-Flag, cDNAs were amplified from Mlph knockout or mutant quail, respectively, and inserted into the pcDNA3.1 vector. To construct quail CD9 expression vector, pmCherry-2xFYVE (Addgene plasmid #140050) was modified. Specifically, pmCherry-2xFYVE was digested using XhoI and KpnI [#R0146 and #R0142, respectively, New England Biolabs (NEB), MA, USA] to excise a FYVE fragment, and the XhoI and KpnI tailed *CD9* PCR product was subsequently ligated using T4 DNA ligase (#M0202, NEB). The sequences of the completely constructed expression vectors were confirmed by sequencing analysis. All oligonucleotide primers used in this study are listed in Table S1.

### Phospholipid-binding assay

To identify which phospholipids bind to Mlph, the PIP Strips membrane (#P23750, ThermoFisher Scientific, Waltham, MA, USA) was blotted with phospholipids using the manufacturer’s instructions. Specifically, the similar protein quantities as those for Flag-fused Mlph WT or mutants (Fig. S1) were incubated with the membrane at 4 °C overnight after blocking with TBS-T (Tris-buffered saline containing 0.1% Tween-20) containing 3% fatty acid-free bovine serum albumin (BSA, #A30075, Mt. Prospect, IL, RPI). After washing with TBS-T, protein binding was visualized using the mouse anti-Flag monoclonal antibody and an HRP-conjugated anti-mouse secondary antibody (#7076, CST). The contents on the membrane were visualized on an X-ray film using ECL plus reagents (#RPN2232, GE Healthcare Biosciences, Pittsburgh, PA, USA). Uncropped blot membranes were shown in Figures S6a and S6b.

### Immunoprecipitation

Flag- or HA-tagged qMlph-WT and -K97E plasmids were co-transfected into the cells to test dimerization of the proteins. For a binding assay between Mlph mutants and Rab27a, 293FT cells were transfected with HA-tagged Rab27a plasmid and/or Flag-tagged several Mlph mutant plasmids: qMlph-WT; -C67A; -C84del; -C92A; -C107A. After 48 h of transfection, cells were scraped on ice in RIPA buffer supplemented with protease inhibitor cocktail (#4693132001, Roche, Switzerland). Cell lysates were then centrifuged at 16,000 × *g* for 10 min at 4 °C. Protein complexes were immunoprecipitated from supernatants with mouse monoclonal anti-Flag G1 Affinity Resin (#TM0634, GeneScript, Piscataway, NJ, USA) or sepharose bead conjugated anti-HA [#3956, Cell Signaling Technology (CST), Danvers, MA, USA], and subsequently visualized using immunoblotting procedures utilizing rabbit monoclonal anti-HA (#3724, CST), or mouse monoclonal anti-Flag (#F1804, Sigma-Aldrich, St. Louis, MO, USA) antibodies, respectively. Uncropped blot membranes were shown in Figure S6c.

### Immunostaining

To investigate localization of Mlph mutants, immunocytochemistry was performed in a quail myoblast cell line (QM7). Vectors containing each of the Mlph mutants were co-transfected with CD9 or FYVE vectors. After 48 h of the incubation, cells were washed, and subsequently permeabilized after fixation with 10% neutral buffered formalin for 1 h. Anti-Flag antibodies were used for detecting Mlph mutants, and Alexa fluor 488 conjugated anti-mouse IgG (#A11011, Invitrogen, Waltham, MA, USA) was used as a secondary antibody. In addition, Phalloidin-CF633 (#00046, Biotium, Hayward, CA, USA) and Lysoview (#70058, Biotium) were used for staining F-actin and lysosome, respectively. There was use of DAPI for nuclei counterstaining.

### Animal care

All animal care and experimental procedures were approved by the Institutional Animal Care and Use Committee at The Ohio State University (Protocol No. 2015A00000013-R1). Experimental quail were maintained at The Ohio State University Poultry Facilities on Columbus, Ohio, USA campus, until birds were euthanized using CO_2_ inhalation that is an approved procedure of the Ohio State IACUC members.

### Generation of Mlph knock-out quail lines

We produced Mlph knock-out quail using procedures described in a previous study we conducted^19^. Specifically, an adenoviral CRISPR/Cas9 vector was constructed and injected into the sub-germinal cavity of the quail blastoderm at Eyal-Giladi and Kochav (EGK) stage XI developmental stage to produce Mlph chimeras. The chimeric birds were bred to produce homogenic Mlph quail.

### Genotype analysis, mutation detection, predictions of protein structure

To confirm homogenic Mlph mutant quail were produced, genomic DNA was extracted from feather germ cells at 3 weeks post-hatching and PCR procedures were performed using DNA Taq-polymerase (#M0273, New England BioLabs, Ipswich, MA) with the condition, 95 °C for 5 min followed by 35 cycles of 95 °C for 30 s, 55 °C for 30 s, 72 °C for 30 s, and 72 °C for 5 min with a primer set^19^. The PCR product was sequenced to determine four different genotypes (WT, −1, −2, or −3 bp). The oligonucleotide primers used for genotyping are listed in Table S1. The protein structures of WT and all mutant Mlph proteins were predicted by using an online-based program, SWISS-MODEL^25^.

### Histological processing and embryonic feather staining

To visualize melanin pigmentation in embryonic feathers at 10 and 15 days after initiation of the incubation of eggs (E10 and E15, respectively), the feathers were fixed in 10% neutral buffered formalin and subsequently processed to embed in paraffin. Paraffin-embedded feathers were cut into 5 μm slices. To highlight the pigmentation characteristics of the feathers, there was Fontana-Masson (FM) staining utilizing the manufacturer’s instruction (#HT-200, Sigma-Aldrich, St. Louis, MO, USA). Nuclear fast red solution (#26078-05, Electron Microscopy Sciences, Hatfield, PA, USA) was used for counterstaining.

### Primary melanocyte culture and immunocytochemistry

To obtain primary melanocytes, skin tissues of birds of Mlph wild-type or mutant quail lines at the E10 were isolated and trypsinized (0.05% Trypsin- EDTA, #15400, Gibco) for 10 min. After filtration through a 70 μm cell strainer (#352350, BD Falcon Franklin Lakes, NJ, USA), cells were washed with PBS and seeded in a melanocyte culture medium containing medium 254 (#M254500, Gibco) supplemented with 1X human melanocyte growth supplement (HMGS, #S0025, Gibco, Grand Island, NY, USA) and 1% antibiotic-antimycotic solution (#15240096, Gibco). For immunocytochemistry, on the day after seeding melanocytes, there was transfection of primary melanocytes with expression vectors containing qMlph-WT or -C84del tagged with Flag. After 48 h, cells were fixed and subsequently stained with anti-Flag and/or phalloidin. To analyze dendricity, length of each dendrite was measured from the edge of the cell body to the end of dendrites in the images obtained after the phalloidin staining, and total dendrite lengths per cell were calculated by determining the sum of each dendrite length within the each cell as previously described^41^.

### Imaging

Imaging of quail body and feathers was performed using a camera (EOS Rebel T7, Canon, Japan). Images of skin and feathers of quail, feather sections, and in vitro cultured cells were obtained using an EVOS cell imaging system (Thermo Fisher Scientific) or a Zeiss LSM 780 confocal microscope.

### Statistical analysis

To analyze the total dendrite length, all data were expressed as means ± SEM. Statistical analyses were performed by the GraphPad Prism software (ver. 6.02) and detailed numbers of cells were described in the figure legends. For one-way ANOVA followed by Tukey’s multiple comparison test, there were considered to be mean differences when there was a *p* < 0.05.

### Reporting summary

Further information on research design is available in the Nature Portfolio Reporting Summary linked to this article.

### Supplementary information

Supplementary Information

Reporting Summary

## Data Availability

The data that support the findings of this study are available from the corresponding author upon reasonable request.
