# Supplementary material for: Melanophilin regulates dendritogenesis in melanocytes for feather pigmentation
Source: Commun Biol. 2024 May 17;7:592. doi: 10.1038/s42003-024-06284-5 (PMC11101434; doi:10.1038/s42003-024-06284-5)
Supplement: Supplementary file 1 — Supplementary Information [file 42003_2024_6284_MOESM1_ESM.pdf]

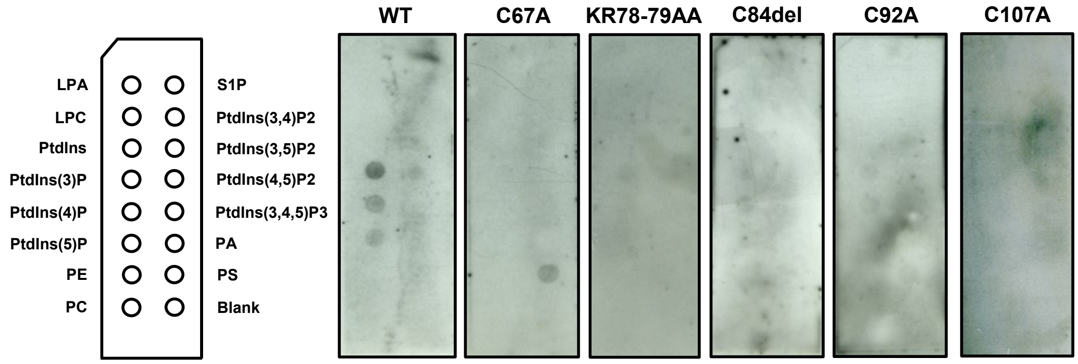

Fig. S1. Phospholipid-binding activity assay. Phospholipids are pre-blotted on the PIP strips membrane. Flag-tagged WT proteins or Flag-tagged Mlph proteins with mutations in the ZnF motif were incubated with the membranes and then immunoblotted using manufacturer instructions. LPA, lysophosphatidic acid; LPC, lysophosphocholine; PtdIns, phosphatidylinositol; PtdIns(3)P, phosphatidylinositol 3-phosphate; PE, phosphatidylethanolamine; PC, phosphatidylcholine; S1P, sphingosine-1-phosphate; PA, phosphatidic acid; PS, phosphatidylserine.

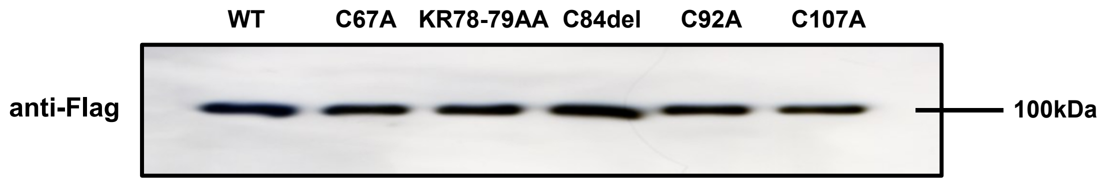

Fig. S2. Western blot analysis to confirm there were similar quantities of Mlph WT and mutant proteins loaded for determination of phospholipid-binding activities.

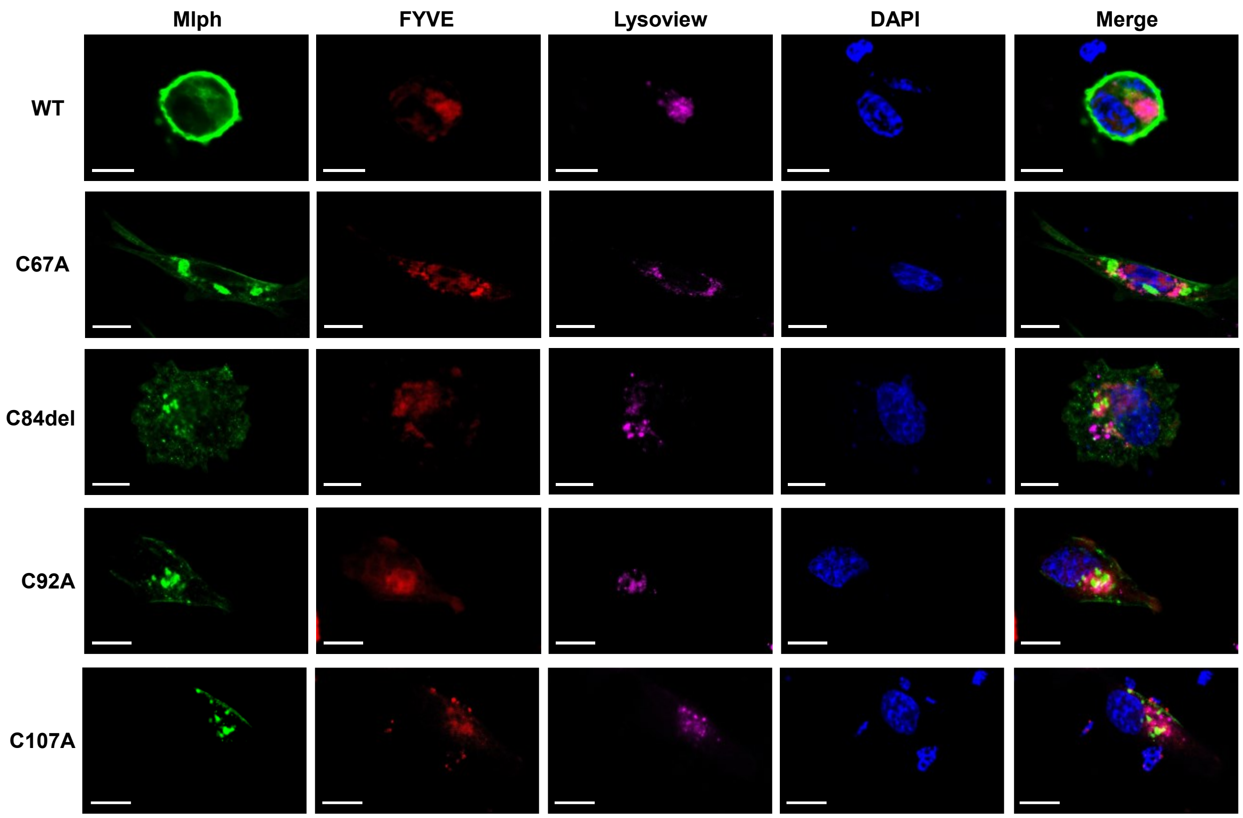

Fig. S3. Disruption of ZnF motif causes mislocalization of Mlph protein to the endosome and lysosome. Flag-tagged Mlph WT or mutants, and pmCherry-FYVE constructs were co-transfected into QM7 cells. After 48 h of transfection, cells were fixed and stained, and subsequently visualized using a confocal microscope. pmCherry-FYVE was used as an endosomal marker, and lysoview for a lysosomal marker. DAPI was used for nuclei staining. Scale bar: 10 μm.

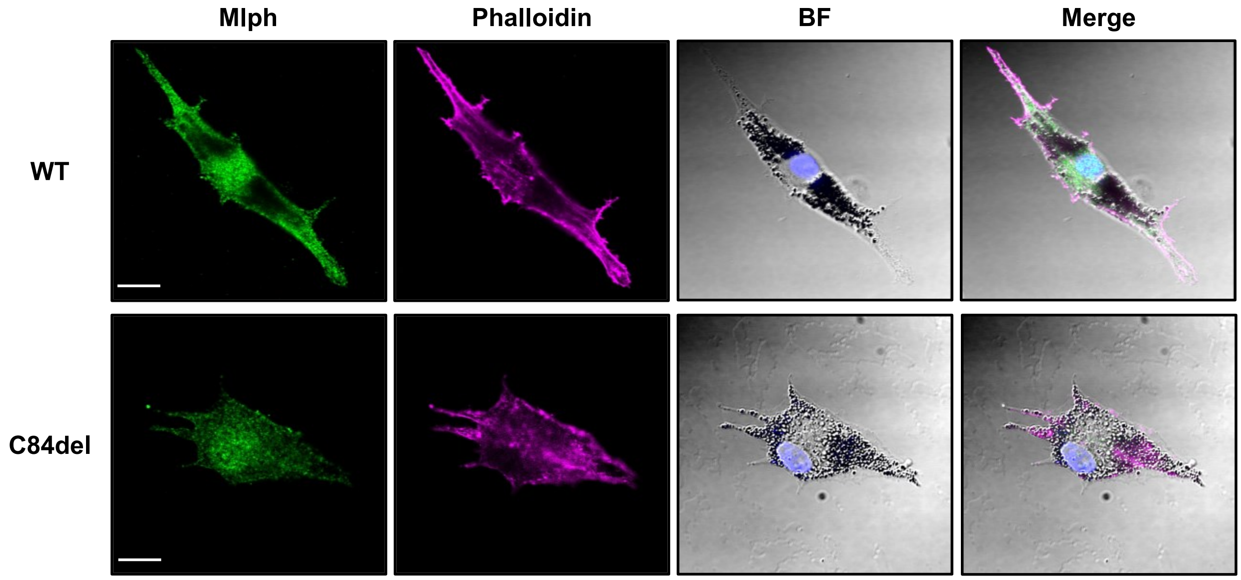

Fig. S4. Cellular localization Mlph proteins when there was primary culturing of skin melanocytes isolated from homozygotic Mlph null quail. Melanocytes were isolated from skin of the -1 bp quail line and transfected the Mlph-WT (WT) or Mlph-C84del (C84del) constructs. After 48 hours of transfection, cells were fixed and stained, and subsequently visualized using a confocal microscope. Nuclei were stained with DAPI, and for actin staining phalloidin was used. Scale bar: 10 μm.

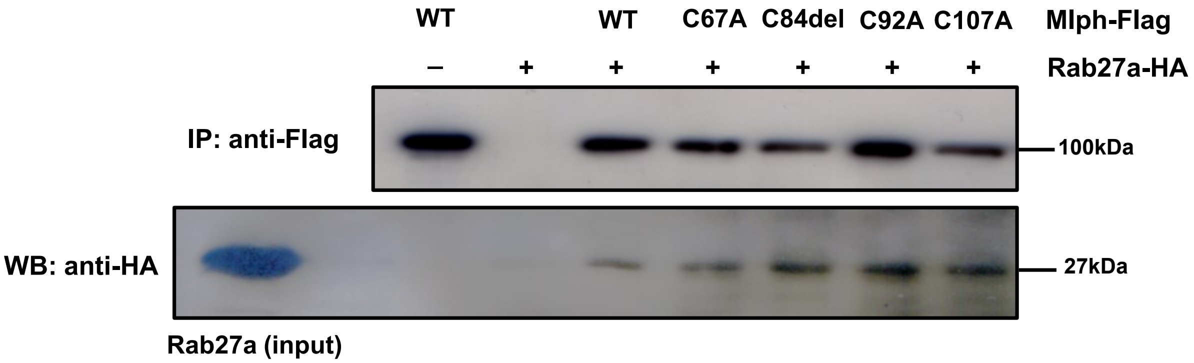

Fig. S5. Association of Mlph mutant proteins with Rab27a. Flag-tagged Mlph WT or mutants were co-transfected with the Rab27a-HA construct into 293FT cells. After 48 h of transfection, proteins were extracted from the cells. Proteins were immunoprecipitated with an anti-Flag antibody and there was an assessment for the interaction between the two proteins using an anti-HA antibody. As an internal control, the relative abundance of Rab27a was determined in the total cell lysate (input).

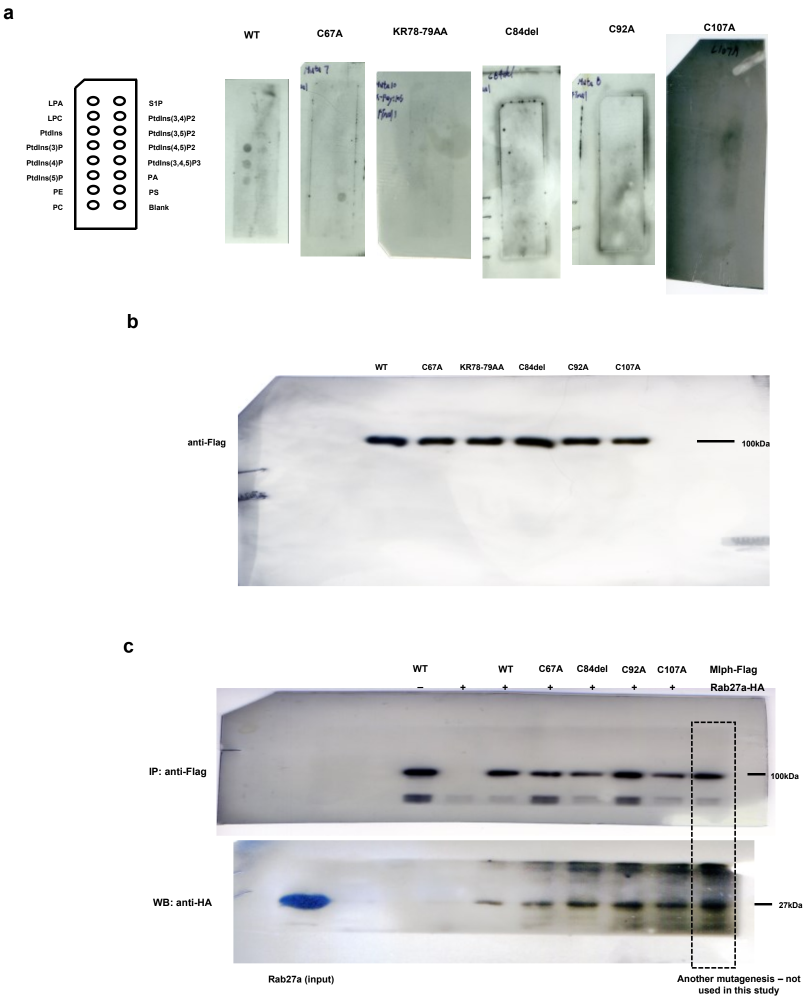

Fig. S6. Uncropped original blots used in Figure S1 (a), S2 (b) and S5 (c).

Table S1. Nucleotide sequences of the primers used.

Purpose	Gene	Primer Sequences
Construct Cloning	qMlph WT-HA	F: 5’ GATGGGGAGGAAGCTGGATC<br>R: 5’ CCTTCAAGCGTAGTCTGGGACGTCGTATGGGTAGGACAGGTGGGTCATCA
	qMlph WT-Flag	F: 5’ GATGGGGAGGAAGCTGGATC<br>R: 5’ TACTGAGACTCTCGAGTCACTTATCGTCATCGTCTTTGTAATCGGACAGGTGGGTCATCACTGGCT
	qRab27a-HA	F: 5’ TATGTCTGATGGGGACTATGATTACCT<br>R: 5’ TACTGAGACTCTCGAGTCAAGCGTAGTCTGGGACGTCGTATGGGTAACAGCCACATTTGCCTTTTTCTTGCT
	qRab27a-Flag	F: 5’ TATGTCTGATGGGGACTATGATTACCT<br>R: 5’ GAGTCACTTATCGTCATCGTCTTTGTAATCACAGCCACATTTGCCTTTTTCTTGCTCCTCACTCA
	qCD9	F: 5’ GATCGGTTCCCTCGAGATGCCTGTCAAAGGAGGCAC<br>R: 5’ TGATCAGTTAGGTACCTTAGACCATTTCTCTGTTTCTGCGG
Mutagenesis	C67A	F: 5’ AAACGTCTCACGCCCTGCAGCCCTTCAAG<br>R: 5’ AAACGTCTCGGGCGTGCACGCAGTGTGTT
	KR78-79AA	F: 5’ AAACGTCTCGCGGCGCAGTGCCTGGATTG<br>R: 5’ AAACGTCTCCGCCGCGCTGTTCAGCAGGAAC
	C92A	F: 5’ AAACGTCTCCGCCAGCCGCTACAACAAG<br>R: 5’ AAACGTCTCCTGGCGCTCTTGCAGGTGTAG
	K97E	F: 5’ AAACGTCTCGAGAGGGAGCAGGGC<br>R: 5’ AAACGTCTCCTCGCGGCGCGGCTGCAGCTC
	C107A	F: 5’ AAACGTCTCCCGCCCGCCTCTCCAGGG<br>R: 5’ AAACGTCTCGGGCGGGATCACAGACCCAG
Quail Genotyping	qMlph	F: 5' CACCAGTCCCACCTGAATGAA<br>R: 5' GGAGAGGCGGCAGGGAT
